# Supplementary material for: A study of evaluating specific tissue oxygen saturation values of gastrointestinal tumors by removing adherent substances in oxygen saturation imaging
Source: PLoS One. 2021 Jan 7;16(1):e0243165. doi: 10.1371/journal.pone.0243165 (PMC7790263; doi:10.1371/journal.pone.0243165)
Supplement: Attachment — Submitted filename: PLOS DOC 20-12467.docx [file pone.0243165.s002.docx]

In this paper Nishihara and colleagues discuss about the efficacy of a new algorithm to evaluate specific tissue oxygen saturation values of gastrointestinal tumors by removing adherent substances in oxygen saturation imaging. The paper topic is potentially interesting because it highlights the possibility of a new, less invasive method to discriminate precancerous or cancerous areas in GI lesions using the superficial tissue oxygen saturation (StO2) and plotting different tumor areas to differentiate neoplastic tissue from other confounding variables such as adherent substances (blood clot ect).

Hovewer even if the topic could be interesting there are some major concerns about the paper content that could be considered before eventual pubblication:

**REVIEWER COMMENTS PAPER : PLOS D-20-12468**

1) In material and methods section there is no specification about tumor type, site, size, grading and general stadiation (T and N stage by CT scan or eus or PET). There is a table but these fundamental informations are not integrated in the text and this can be misleading. Moreover the authors do not specify if this heterogeneous cluster of lesions can be matched or could affect the results they discuss later in the text.
2) the entire result section is almost confusing. It's not specify how the authors utilize the algorithm, how they decide to apply it (indications by the Fujifilm i.e?) and in general the results are showed using the figures but they are not clearly explained so it's difficult to read this part.
3) the abstract section has to be re written starting from the over mentioned point.
4) the discussion is also not to clear because the authors do not specify how the results can be used in the clinical practice probably it's due to the unclear results section too.
