# Supplementary material for: A study of evaluating specific tissue oxygen saturation values of gastrointestinal tumors by removing adherent substances in oxygen saturation imaging
Source: PLoS One. 2021 Jan 7;16(1):e0243165. doi: 10.1371/journal.pone.0243165 (PMC7790263; doi:10.1371/journal.pone.0243165)
Supplement: Attachment — Submitted filename: Response to Reviewers.docx [file pone.0243165.s003.docx]

20th, September, 2020

Joerg Heber

Editor-in-Chief

PLOS ONE

We wish to re-submit manuscript titled “A study evaluating specific tissue oxygen saturation values of gastrointestinal tumors by removing adherent substances in oxygen saturation imaging” The manuscript ID is PONE-D-20-12468.

We thank you for giving us the opportunity to revise the manuscript. We have carefully studied all the suggestions. The manuscript has benefited from these insightful suggestions. We look forward to working with you and the reviewers to move this manuscript closer to publication in PLOS ONE. The manuscript has been rechecked and the necessary changes have been made in accordance with the reviewer’s suggestions.

The responses to all comments have been prepared and given below.

Thank you for your consideration. We look forward to hearing from you.

Sincerely,

Corresponding author: Tomonori Yano

Department of Gastroenterology and Endoscopy, National Cancer Center Hospital East.

6-5-1, Kashiwanoha, Kashiwa, 277-8577, Japan.

**Reply for Reviewers’ comments**

**Reviewer #1**

1) In material and methods section there is no specification about tumor type, site, size, grading and general stadiation (T and N stage by CT scan or eus or PET). There is a table but these fundamental informations are not integrated in the text and this can be misleading. Moreover the authors do not specify if this heterogeneous cluster of lesions can be matched or could affect the results they discuss later in the text.

Answer:

Thank you for your invaluable suggestion. We have added information on lymph node metastasis, distant metastasis, and TNM Staging of cases used in the analysis in revised Table1. We think that the clinical pathological features of Advanced tumor are likely to affect the Sto2 values of tumors. As the reviewer pointed out, we evaluated the small samples of heterogeneous cluster to build the algorism, and it is hard to deny the possibility to affect the results. However, here is no significant difference in clinical pathological characteristics between the case used in the construction of Algorithm and the case used for its verification as shown in Table 1.

This point was added to the discussion section as a limitation.

2) the entire result section is almost confusing. It's not specify how the authors utilize the algorithm, how they decide to apply it (indications by the Fujifilm i.e?) and in general the results are showed using the figures but they are not clearly explained so it's difficult to read this part.

Answer:

We agree with your assessment. I modified the explanation of the result section to make it easier to understand.

The following points are emphasized; (1) It was proved by constructing a discriminating algorithms with high accuracy rate that it is possible to discriminate between tumors and their adherent substances that can be discriminated by endoscopic images of white light. (2) It was suggested that the current OS imaging may be strongly influenced by adherent substances by examined StO2 values in the three regions of the whole tumor region including adherent tumors, only tumor region, and only adherent substances region defined by the discriminating algorithms.

3) the abstract section has to be re written starting from the over mentioned point.

Answer:

We agree with your assessment and revised the abstract section to reflect what we would like to mention.

4) the discussion is also not to clear because the authors do not specify how the results can be used in the clinical practice probably it's due to the unclear results section too.

Answer:

We agree with you and added following point in discussing section ; The construction of the discriminating algorithms with high accuracy rate suggests that it is possible to evaluate tumor-specific StO2 values by removing the StO2 information in the regions defined by the algorithm as adherent substances.

**Reviewer #2**

As mentioned by the authors, the main limits of this study are the limited number of patients and that the annotation point were selected randomly and by only two endoscopist. This could affect the reliability of this algorithm, that should be validated in a larger multi-center study in order to evaluate the reproducibility of this algorithm.

However, this study try to overcome the limitations of this new technology, which could have interesting applications in gastrointestinal tumors in the next future.

I suggest to the authors to largely explain in the discussion the possible future applications on this technology and largely explain the differences with other diagnostic imaging methods.

Answer:

Thank you for your valuable feedback. As you pointed out, this study is based on the premise that only two endoscopists perform annotations and that the annotates can correctly distinguish between tumors and adherent substances. In the future, I think we should consider a study in a multi-center study for a more accurate examination.

Regarding the future outlook, constructing more precise algorithm make it possible to remove noises on the tumor surface. If the endoscopic system of OS imaging that implements the noise correction in real time by the algorithms is constructed, it will be possible to measure the StO2 values of the tumor in real time. This may be a biomarker for determining the therapeutic effect during or after treatment in treatments such as PDT that do not remove the tumor.

I added this point to the discussion part.
